# The association among uric acid, microalbumin and estimated glomerular filtration rate in hypertensive patients: a case control study

**DOI:** 10.1186/s12872-023-03085-2

**Published:** 2023-02-05

**Authors:** Hongda Chou, Maoti Wei, Hongxia Chen, Yuanyuan Xu, Leilie Shi, Jiajia Duan, Linlin Li, Ning Yang, Yuming Li

**Affiliations:** 1 Department of Graduate School, Tianjin Medical University, Tianjin, 300051 China; 2 Department of Hypertension, TEDA International Cardiovascular Hospital, Tianjin, 300457 China; 3 Center for Clinical Epidemiology, TEDA International Cardiovascular Hospital, Tianjin, 300457 China; 4 Intensive Care Unit, TEDA International Cardiovascular Hospital, Tianjin, 300457 China; 5 Department of Cardiology, TEDA International Cardiovascular Hospital, Tianjin, 300457 China

**Keywords:** Uric acid, Hypertension, Microalbumin, Estimated glomerular filtration rate, Mediating effect

## Abstract

**Objective:**

To estimate the relationship among uric acid (UA), 24-h microalbumin (24 h-MAU) and estimated glomerular filtration rate (eGFR) in hypertensive patients.

**Method:**

The study enrolled adult patients hospitalized in TEDA International Cardiovascular Hospital. The study was used to explore the correlation among UA, 24 h-MAU and eGFR. Univariate analysis was used to compare continuous or categorical data groups according to data type. Multivariate analysis was used to explore the correlation among UA, Log 24 h-MAU and eGFR by linear regression, and the relationship among UA, 24 h-MAU ≥ 30 mg/24 h (increased 24 h-MAU) and eGFR < 90 ml·min^−1^·1.73 m^−2^ (mildly decreased eGFR) by logistic regression. Mediation effect analysis was used to explore the mediating effect of increased 24 h-MAU between UA and mildly decreased eGFR. Subgroup analysis was used to investigate the correlation among UA, 24 h-MAU and eGFR in different gender.

**Result:**

Seven hundred and thirty-three inpatients were enrolled in the study, including 257 patients with hyperuricemia. The level of UA was 377.8 ± 99.9 μmol/L in all patients enrolled, and it was about 50.1% higher in hyperuricemia group (482.3 ± 58.8 μmol/L vs. 321.4 ± 63.5 μmol/L, *P* < 0.001). The prevalence of hyperuricemia was 35.1% (*95%CI* 31.6–38.5%). The univariate regression analysis showed that UA was significant related to Log 24 h-MAU, increased 24 h-MAU, eGFR and mildly decreased eGFR. After adjusted confounding factors, UA was significant related to Log 24 h-MAU (*β* = 0.163, *P* < 0.001), eGFR (*β* = − 0.196, *P* < 0.001), increased 24 h-MAU (quantitative analysis: *OR* = 1.045, *95%CI* 1.020–1.071, *P* < 0.001; qualitative analysis: *OR* = 2.245, *95%CI* 1.410–3.572, *P* = 0.001), but had no significant relationship with mildly decreased eGFR. Mediating effect analysis showed that increased 24 h-MAU partially mediated the relationship between UA and mildly decreased eGFR (relative indirect effect: 25.0% and 20.3% in quantitative analysis and qualitative analysis respectively). In the subgroup analysis, the results were stable and similar to the analysis for entry patients.

**Conclusion:**

The prevalence of hyperuricemia was higher in hypertensive inpatients. UA was strongly associated with Log 24 h-MAU, eGFR and increased 24 h-MAU, while the correlation with mildly decreased eGFR was affected by multiple factors. And increased 24 h-MAU might be the intermediate factor between UA and mildly decreased eGFR.

**Supplementary Information:**

The online version contains supplementary material available at 10.1186/s12872-023-03085-2.

## Introduction

Hypertension is one of the most common chronic diseases and the most important risk factor for cardiovascular diseases in the world [1]. The results of the Hypertension Survey in China showed that it was about 23.2% (240 million) adults suffered from hypertension from 2012 to 2015 [2]. Hypertension is often associated with a variety of metabolic disorders, including glucose metabolism disorders, lipid metabolism disorders and uric acid (UA) metabolism disorders. Hyperuricemia is a metabolic disorder syndrome caused by purine metabolism disorder [3]. The incidence of hyperuricemia was increasing year by year, and showed the characteristics that male was higher than female and South was higher than North [4]. The incidence of hyperuricemia was 10.3% [5] to 17.2% [6] in Chinese hypertensive patients.

Many evidences show that kidney was one of the main target organ damages of hypertension. Meanwhile, elevated UA was also an important risk factor for chronic kidney disease (CKD) [7]. The previous study had shown that hypertension and hyperuricemia were not only independently related to kidney damage, but also had a certain synergistic effect on kidney damage [5]. In view of the high incidence of hyperuricemia in hypertensive patients, paying attention to kidney damage would play an important role in the early treatment and improving outcomes in hypertensive patients with hyperuricemia.

The factors of 24-h microalbumin (24 h-MAU) and estimated glomerular filtration rate (eGFR) were used to evaluate the state of kidney damage. Among them, 24 h-MAU was commonly used to evaluate the degree of early kidney damage, and eGFR was a direct evaluation index of kidney function. To further clarify the potential role of UA in kidney damage in hypertensive patients, this study used the data of hospitalized hypertensive patients to explore the correlation among UA, 24 h-MAU and eGFR.

## Method

### Study population

The hospitalized hypertensive patients were selected in TEDA International Cardiovascular Hospital from April 2020 to May 2022. Inclusion criteria included: (1) Age ≥ 18 years old; (2) Meet the diagnostic criteria of hypertension; (3) Sign the informed consent for admission. Exclusion criteria included: (1) Patients with definite secondary hypertension factors; (2) Patients with severe cardio-cerebrovascular complications (acute heart failure, acute myocardial infarction, acute cerebral infarction, acute cerebral hemorrhage, etc.); (3) Uncomplete UA test; (4) Patients with previous kidney diseases (renal surgery, congenital renal structural malformations, glomerulonephritis, nephrotic syndrome, polycystic kidney disease, etc.).

This study was conducted in accordance with the ethical standards of the declaration of Helsinki. It was approved by the Ethics Committee of TEDA International Cardiovascular Hospital and exempt from the informed consent.

### General information collection

The patients' age, history of hypertension, past medical history, height, weight and other information were collected. All history taking and physical examination were performed by hypertension specialists.

### Laboratory data collection

The data of white blood cell, red blood cell, hemoglobin, fasting blood glucose, 2-hour blood glucose, total cholesterol, triglyceride, high-density lipoprotein cholesterol, low-density lipoprotein cholesterol, creatinine, UA, 24-h urinary sodium and 24 h-MAU were collected. The above blood tests and urine related tests were completed by the laboratory examination department of our hospital.

### Ambulatory blood pressure measurement

Ambulatory blood pressure was used to evaluate the blood pressure level of hospitalized patients. Choose the appropriate size cuff according to the arm circumference. The duration of ambulatory blood pressure monitoring should be no less than 24 h. The recommended time interval for automatic blood pressure measurement is: every 15 min in the daytime and every 30 min in the night. The effective reading ≥ 70% should be taken to obtain the reading, and the reading should be ≥ 20 times during the day and ≥ 7 times at night. The operation is performed by trained medical personnel.

### Calculation of eGFR

The modified simplified MDRD equation was used to calculate eGFR [8]. Modified simplified MDRD equation: eGFR = 175 × Scr^−1.234^ × Age^−0.179^[× 0.79 (female)].

### Diagnosis definition

Hyperuricemia was defined as UA > 420 μmol/L (7 mg/dl) for male and > 360 μmol/L (6 mg/dl) for female. Mildly decreased eGFR was defined as eGFR < 90 ml·min^−1^·1.73 m^−2^; decreased eGFR was defined as eGFR < 60 ml·min^−1^·1.73 m^−2^; increased 24 h-MAU was defined as 24 h-MAU ≥ 30 mg/24 h.

### Statistical analysis

**Fig. 1:**
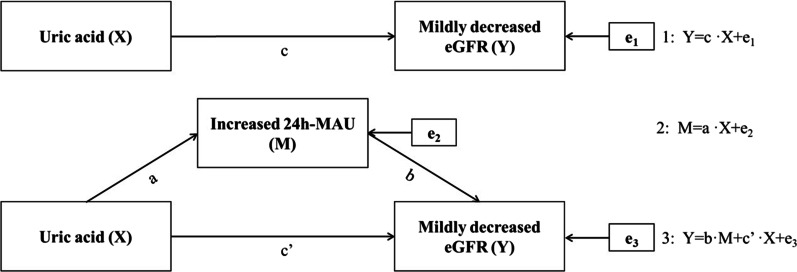
Establishment of mediating effect analysis model of increased 24 h-MAU (M) between UA (X) and mildly decreased eGFR (Y)

The historical case control study was used for data collation and analysis. Univariate analysis was performed to compare continuous or categorical data between groups according to data type. Categorical data were represented by use numbers (%), and comparisons between groups were performed using the χ^2^ test. Continuous variables conforming to normal distribution were expressed as mean ± standard (x ± s), and independent sample* t* test or *t'* test was used for comparison between groups. The median (*P*_25_–*P*_75_) was used to express the skewed distribution of continuous variables, and the nonparametric test was used for comparison between groups. Logistic regression was used to explore the correlation among UA, increased 24 h-MAU and mildly decreased eGFR and linear regression analysis was used to explore the correlation among UA, Log 24 h-MAU and eGFR. Forward conditional was used to screen variables for multivariate analysis. The probability of variable entering the equation was 0.05, and the probability of variable removal was 0.10. Subgroup analysis was set up to verify the relationship among UA, 24 h-MAU and eGFR in different gender. Mediating effect analysis [9] was used to explore the intermediate effect of increased 24 h-MAU on UA and mildly decreased eGFR. UA was taken as independent variable (X) with continuous variables and categorical variables respectively, increased 24 h-MAU as mediating variable (M), and mildly decreased eGFR as dependent variable (Y). Three Logistic regression equations were established (Fig. 1). The asymmetric *95% CI* of *Z*_*a*_ × *Z*_*b*_ was calculated by *Prodclin2*, and it was considered statistically significant if the *95%CI* did not contain 0. All of data were analyzed and processed by professional statistical analysts using SPSS Version 24.00 (Armonk, NY: IBM Corp). *P* < 0.05 was considered statistically significant.

## Results

### Population characteristics

**Fig. 2:**
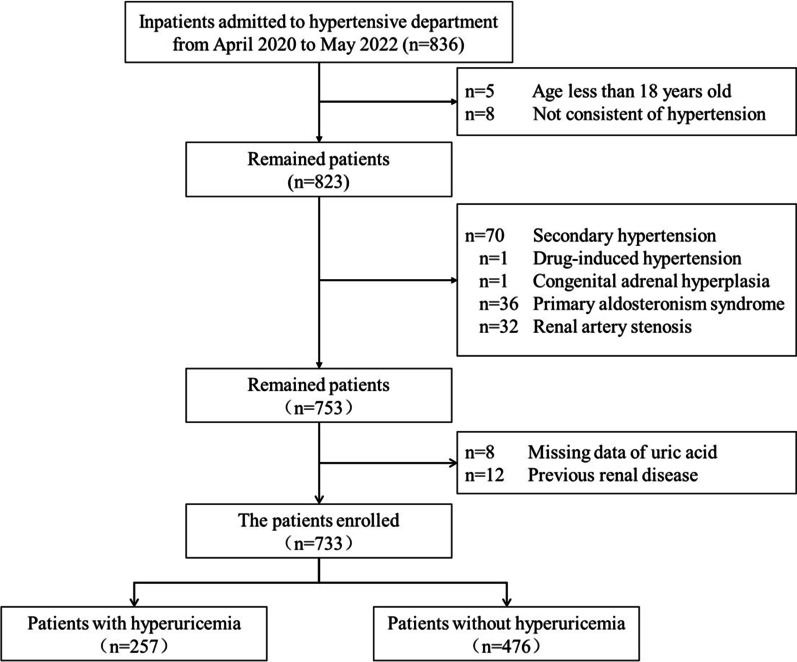
Flow chart of study population enrollment

A total of 733 hypertensive patients were included in the study (Fig. 2), with an average age of 42.6 ± 12.3 years and 72.4% of them were male. There were 257 patients with hyperuricemia, and the incidence of hyperuricemia was 35.1% (*95%CI* 31.6–38.5%), and 85.2% of them were male. The level of UA was 377.8 ± 99.9 μmol/L in hospitalized hypertensive patients. Among them, the hyperuricemia group was 482.3 ± 58.8 μmol/L, which was about 50.1% higher than non-hyperuricemia group (321.4 ± 63.5 μmol/L) (*P* < 0.001). The prevalence of diabetes, coronary heart disease and cerebrovascular disease were low (all < 10%).

The age was 44.6 ± 12.7 years and 38.8 ± 10.6 years in non-hyperuricemia group and hyperuricemia group respectively. The degree of hypertension, body mass index (BMI), triglyceride, creatinine, mean systolic blood pressure, and mean diastolic blood pressure in hypertension patients with hyperuricemia were significantly higher than those in patients without hyperuricemia (*P* < 0.05) (Table 1).

**Table 1:** The characteristics of the study population

	Non-hyperuricemia group (n = 476)	Hyperuricemia group (n = 257)	*P*
Age, years	44.6 ± 12.7	38.8 ± 10.6	< 0.001
Male, n (%)	312 (65.5)	219 (85.2)	< 0.001
BMI, kg/m^2^	26.1 ± 3.6	28.7 ± 4.0	< 0.001
Duration of hypertension, months	24 (3, 72)	24 (4, 60)	0.698
Grade of hypertension			0.071
Non-grade 3 hypertension, n (%)	180 (37.8)	80 (31.1)	
Grade 3 hypertension, n (%)	296 (62.2)	177 (68.9)	
Smoking, n (%)	167 (35.1)	103 (40.1)	0.181
Alcohol intake, n (%)	72 (15.1)	40 (15.6)	0.875
Diabetes mellites. n (%)	37 (7.8)	11 (4.3)	0.068
CHD, n (%)	25 (5.3)	4 (1.6)	0.014
Cerebrovascular disease, n (%)	22 (4.6)	3 (1.2)	0.014
Antihypertension drugs intake			
ACEI/ARB, n (%)	175 (40.4)	101 (43.5)	0.437
β-blocker, n (%)	70 (16.2)	26 (11.2)	0.083
CCB, n (%)	182 (42.0)	99 (42.7)	0.873
Diuretic, n (%)	47 (10.9)	26 (11.2)	0.890
α-blocker, n (%)	12 (2.8)	10 (4.3)	0.290
MRA, n (%)	2 (0.5)	2 (0.9)	0.528
WBC, 10^9^/L	6.2 ± 1.6	6.6 ± 1.7	0.001
RBC, 10^12^/L	4.8 ± 0.5	5.0 ± 0.4	< 0.001
HGB, g/L	145.7 ± 16.0	152.0 ± 13.4	< 0.001
Fasting glucose, mmol/L	5.2 ± 1.3	5.3 ± 1.4	0.322
2-h glucose, mmol/L	8.8 ± 2.9	8.7 ± 2.5	0.601
TC, mmol/L	4.6 ± 0.9	4.7 ± 0.9	0.025
TG, mmol/L	1.6 ± 1.1	2.3 ± 1.5	< 0.001
HDL-C, mmol/L	1.1 ± 0.3	1.1 ± 0.3	< 0.001
LDL-C, mmol/L	3.0 ± 0.8	3.1 ± 0.8	0.055
Cr, mg/dL	0.7 ± 0.2	0.8 ± 0.2	< 0.001
UA, μmol/L	321.4 ± 63.5	482.3 ± 64.5	< 0.001
Daytime mean SBP, mmHg	134.6 ± 15.2	139.9 ± 16.9	< 0.001
Nighttime mean SBP, mmHg	123.7 ± 17.2	128.4 ± 19.1	0.001
24 h mean SBP, mmHg	132.0 ± 15.0	137.1 ± 16.8	< 0.001
Daytime mean DBP, mmHg	82.6 ± 13.2	86.7 ± 12.8	< 0.001
Nighttime mean DBP, mmHg	73.4 ± 13.2	77.8 ± 13.6	< 0.001
24 h mean DBP, mmHg	80.4 ± 12.8	84.6 ± 12.6	<0.001
24 h urinary sodium, mmol/24 h	146.5 ± 63.2	156.5 ± 63.7	0.047
24 h-MAU, mg/24 h	13.9 (8.4, 27.8)	21.0 (1.0, 44.5)	< 0.001
≥ 30 mg/24 h, n (%)	104 (23.3)	94 (37.8)	< 0.001
eGFR, ml·min^−1^·1.73 m^−2^	131.6 ± 29.7	119.8 ± 28.5	< 0.001
< 90 ml·min^−1^·1.73 m^−2^, n (%)	26 (5.5)	31 (12.1)	0.001
< 60 ml·min^−1^·1.73 m^−2^, n (%)	2 (0.4)	3 (1.2)	0.241

Categorical data were represented by use numbers (%). Continuous variables conforming to normal distribution were expressed as mean ± standard (x ± s). The median (*P*_25_–*P*_75_) was used to express the skewed distribution of continuous variables.

*BMI*: body mass index; *ACEI/ARB*: angiotensin-converting enzyme inhibitor/angiotensin receptor inhibitor; *CCB*: calcium channel blockers; *MRA*: mineralocorticoid receptor antagonists; *WBC*: white blood cell; *RBC*: red blood cell; *HGB*: hemoglobin; *TC*: total cholesterol; *TG*: triglycerides; *UA*: uric acid; *SBP*: systolic blood pressure; *DBP*: diastolic blood pressure; *24 h-MAU*: 24-h microalbuminuria; *eGFR*: estimated glomerular filtration rate

The rate of increased 24 h-MAU was 27.0% in the study population. The rate of mildly decreased eGFR was 7.8% and decreased eGFR was 0.7%. Further analysis showed that the hyperuricemia group had more severe kidney damage than those in non-hyperuricemia group (Table 1), such as the level of 24 h-MAU (21.0 mg/24 h vs. 13.9 mg/24 h, *P* < 0.001) and the prevalence of increased 24 h-MAU (37.8% vs. 23.3%, *P* < 0.001). The level of eGFR (131.6 ± 29.7 ml·min^−1^·1.73 m^−2^ vs. 119.8 ± 28.5 ml·min^−1^·1.73 m^−2^, *P* < 0.001) was decreased and the prevalence of mildly decreased eGFR (5.5% vs. 12.1%, *P* = 0.001) was increased in hypertensive patients with hyperuricemia (Table 1).

### The analysis for the relationship among UA, 24 h-MAU and eGFR

Linear regression analysis was set up to estimate the relationship among UA, Log 24 h-MAU and eGFR. The results of univariate linear analysis indicated that UA was significant related to Log 24 h-MAU (*β* = 0.210, *P* < 0.001) and eGFR (*β* = − 0.286, *P* < 0.001) (Additional file 1: Table S1). After adjusting the confounding factors, UA still had significant relationship with Log 24 h-MAU (*β* = 0.163, *P* < 0.001) and eGFR (*β* = − 0.196, *P* < 0.001). Other related factors for Log 24 h-MAU were duration of hypertension, grade 3 hypertension and fasting glucose. And other related factors for eGFR were age, gender, ACEI/ARB intake, RBC and Log 24 h-MAU (Table 2).

**Table 2:** The multivariate linear regression analysis of Log 24 h-MAU and eGFR

Variates	Log 24 h-MAU					Variates	eGFR				
	B	SE	*β*	*t*	*P*		B	SE	*β*	*t*	*P*
UA (every increased 10 μmol/L)	0.007	0.002	0.163	3.549	< 0.001	UA (every increased 10 μmol/L)	− 0.573	0.126	− 0.196	− 4.531	< 0.001
Duration of hypertension						Age	− 0.796	0.106	− 0.310	− 7.522	< 0.001
Less than 1 year	Reference					Gender (male)	15.316	3.167	0.226	4.836	< 0.001
1–5 years	0.057	0.047	0.062	1.198	0.232	ACEI/ARB intake	− 6.157	2.301	− 0.102	− 2.676	0.008
5–10 years	0.170	0.061	0.141	2.786	0.006	RBC	− 6.972	2.905	− 0.105	− 2.400	0.017
More than 10 years	0.089	0.062	0.073	1.432	0.153	Log 24 h-MAU	− 5.136	2.392	− 0.081	− 2.147	0.032
Grade 3 hypertension	0.131	0.043	0.144	3.052	0.002						
Fasting glucose	0.087	0.027	0.147	3.176	0.002						

*24 h-MAU*: 24-h microalbuminuria; *eGFR*: estimated glomerular filtration rate; *UA*: uric acid; *ACEI/ARB*: angiotensin-converting enzyme inhibitor/angiotensin receptor inhibitor; *RBC*: red blood cell

Logistic regression analysis was set up to verify the correlation among UA, increased 24 h-MAU and mildly decreased eGFR. The results of univariate logistic analysis showed that UA (every increased 10 μmol/L) and hyperuricemia were significant associated to increased 24 h-MAU (quantitative analysis: *OR* = 1.046, *95%CI* 1.028–1.064, *P* < 0.001; qualitative analysis: *OR* = 2.000, *95%CI* 1.427–2.803, *P* < 0.001) and mildly decreased eGFR (quantitative analysis: *OR* = 1.055, *95%CI* 1.028–1.084, *P* < 0.001; qualitative analysis: *OR* = 2.374, *95%CI* 1.376–4.095, *P* = 0.002) (Additional file 1: Table S2). After adjusting confounding factors, UA was significant related to increased 24 h-MAU (quantitative analysis: *OR* = 1.045, *95%CI* 1.020–1.071, *P* < 0.001; qualitative analysis: *OR* = 2.245, *95%CI* 1.410–3.572, *P* = 0.001). However, UA showed no significant relationship with mildly decreased eGFR. The related factors of mildly decreased eGFR were age, red blood cell, fasting glucose, nighttime mean diastolic blood pressure and increased 24 h-MAU (Table 3).

**Table 3:** The multivariate logistic regression analysis of increased 24 h-MAU and mildly decrease eGFR

Model^*^	Variates	Increased 24 h-MAU						Variates	Mildly decreased eGFR					
		B	SE	Wald χ^2^	*P*	OR	95%CI		B	SE	Wald χ^2^	*P*	OR	95%CI
1	UA (every increased 10 μmol/L)	0.044	0.013	12.573	< 0.001	1.045	1.020–1.071	Age	0.050	0.021	5.599	0.018	1.051	1.009–1.096
	Duration of hypertension			11.408	0.010			RBC	1.023	0.451	5.141	0.023	2.783	1.149–6.741
	Less than 1 year	Reference						Fasting glucose	0.468	0.230	4.129	0.042	1.597	1.017–2.509
	1–5 years	0.181	0.298	0.366	0.545	1.198	0.667–2.150	Nighttime mean DBP	0.028	0.014	4.288	0.038	1.029	1.002–1.057
	5–10 years	1.041	0.352	8.740	0.003	2.831	1.420–5.644	Increased 24 h-MAU	0.705	0.390	3.266	0.071	2.023	0.942–4.343
	More than 10 years	0.745	0.355	4.399	0.036	2.106	1.050–4.226							
	Grade 3 hypertension	0.893	0.278	10.313	0.001	2.442	1.416–4.211							
	Fasting glucose	0.344	0.155	5.256	0.022	1.425	1.053–1.930							
	Daytime mean SBP	0.016	0.007	4.756	0.029	1.016	1.002–1.030							
2	Hyperuricemia	0.809	0.237	11.634	0.001	2.245	1.410–3.572	Age	0.050	0.021	5.599	0.018	1.051	1.009–1.096
	Duration of hypertension			11.981	0.007			RBC	1.023	0.451	5.141	0.023	2.783	1.149–6.741
	Less than 1 year	Reference						Fasting glucose	0.468	0.230	4.129	0.042	1.597	1.017–2.509
	1–5 years	0.154	0.297	0.270	0.603	1.167	0.652–2.090	Nighttime mean DBP	0.028	0.014	4.288	0.038	1.029	1.002–1.057
	5–10 years	1.049	0.350	8.987	0.003	2.856	1.438–5.672	Increased 24 h-MAU	0.705	0.390	3.266	0.071	2.023	0.942–4.343
	More than 10 years	0.756	0.354	4.554	0.033	2.130	1.064–4.266							
	Grade 3 hypertension	0.887	0.279	10.073	0.002	2.428	1.404–4.198							
	Fasting glucose	0.392	0.155	6.431	0.011	1.480	1.093–2.004							
	Daytime mean DBP	0.022	0.010	5.339	0.021	1.023	1.003–1.042							

*Model 1: included UA (every increased 10 μmol/L) as argument. Model 2: included hyperuricemia as argument.

*24 h-MAU*: 24-h microalbuminuria; *increased 24 h-MAU*: 24 h-MAU ≥ 30 mg/24 h; *eGFR*: estimated glomerular filtration rate; mildly decreased *eGFR*: eGFR < 90 ml·min^−1^·1.73 m^−2^; *RBC*: red blood cell; *SBP*: systolic blood pressure; *DBP*: diastolic blood pressure

### Mediating effect of increased 24 h-MAU on the relationship between UA and decreased eGFR

**Fig. 3:**
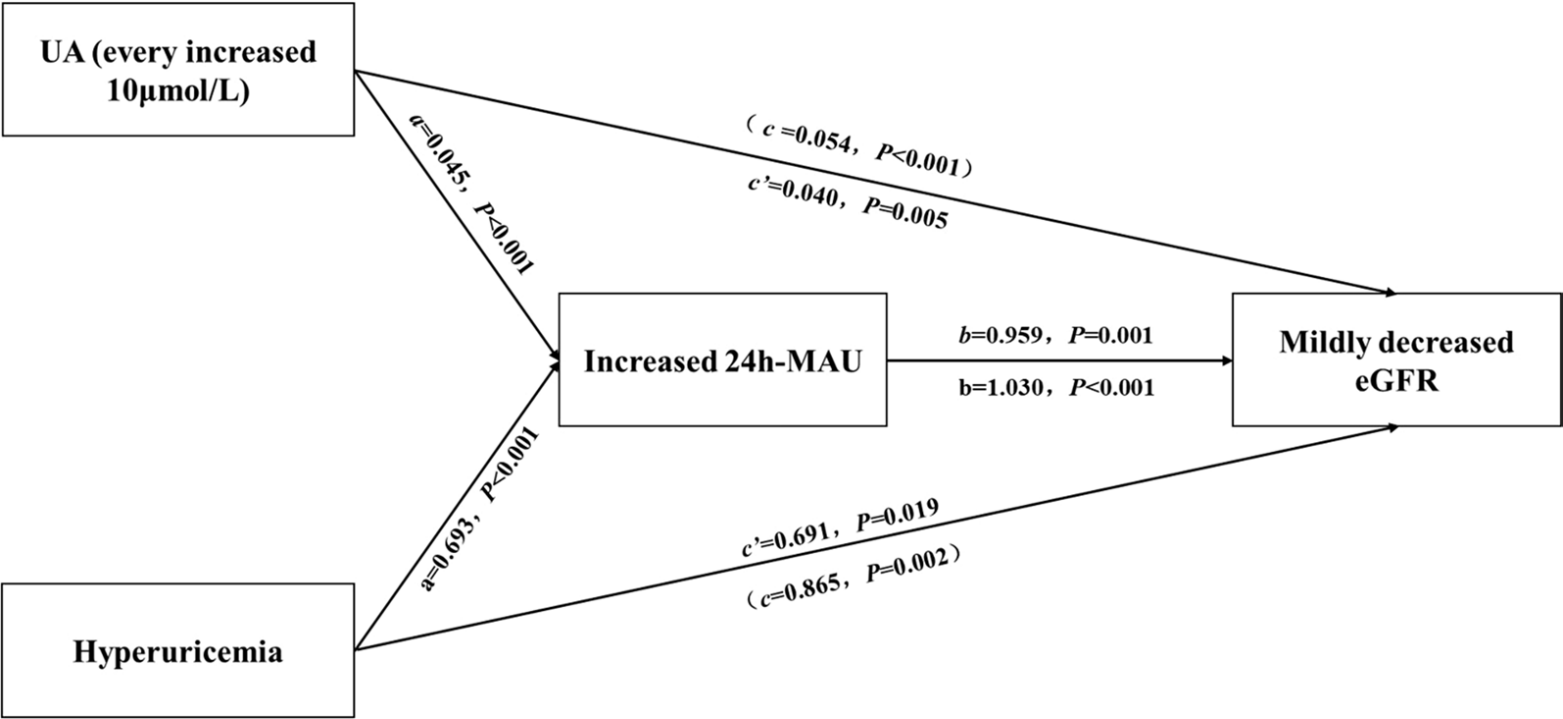
Analysis for the mediating effect of increased 24 h-MAU on the relationship between UA and mildly decreased eGFR

Mediating effect analysis was used to further explore whether increased 24 h-MAU was a mediating variable between UA and mildly decreased eGFR (Fig. 3). Quantitative and qualitative data were used as independent variables for UA. The results showed that increased 24 h-MAU had a significant mediating effect on the relationship between UA and mildly decreased eGFR (quantitative analysis: *95%CI*_*Za*×*Zb*_: 0.015–0.079; qualitative analysis: *95%CI*_*Za*×*Zb*_: 0.170–1.162), and it showed a partially mediating effect (relative indirect effect: 25.0% and 20.1% in quantitative analysis and qualitative analysis respectively) (Table 4).

**Table 4:** The mediating effect of increased 24 h-MAU on the relationship between UA and mildly decreased eGFR

Equation	Independent variable	Dependent variable	B	SE	*P*	*OR*(*95%CI*)	*Z*_*a*_ × *Z*_*b*_	Asymmetric *95% CI*_*Za×Zb*_	Mediating effect (%)
Model 1*									
1)c	UA (every increased 10 μmol/L)	Mildly decreased eGFR	0.054	0.014	< 0.001	1.055 (1.028–1.084)	16.037	0.015–0.079	25.0
2)a	UA (every increased 10 μmol/L)	Increased 24 h-MAU	0.045	0.009	< 0.001	1.046 (1.028–1.064)			
3)b	Increased 24 h-MAU	Mildly decreased eGFR	0.959	0.299	0.001	2.609 (1.453–4.683)			
c’	UA (every increased 10 μmol/L)		0.040	0.014	0.005	1.041 (1.012–1.070)			
Model 2*									
1)c	Hyperuricemia	Mildly decreased eGFR	0.865	0.278	0.002	2.374 (1.376–4.095)	14.115	0.170–1.162	20.1
2)a	Hyperuricemia	Increased 24 h-MAU	0.693	0.172	< 0.001	2.000 (1.427–2.803)			
3)b	Increased 24 h-MAU	Mildly decreased eGFR	1.030	0.294	< 0.001	2.800 (1.574–4.980)			
c’	Hyperuricemia		0.691	0.294	0.019	1.996 (1.121–3.552)			

*Model 1: included UA (every increased 10 μmol/L) as argument. Model 2: included hyperuricemia as argument. 24 h-MAU: 24-h microalbuminuria; increased 24 h-MAU: 24 h-MAU ≥ 30 mg/24 h; *eGFR*: estimated glomerular filtration rate; mildly decreased *eGFR*: eGFR < 90 ml·min^−1^·1.73 m^−2^

### Subgroup analysis of each gender

As gender might be an important influence factor for UA, multivariate linear and logistic regression analysis were used to set up subgroup analysis for each gender.

In linear regression analysis, the model enrolled age, BMI, duration of hypertension, grade of hypertension, smoking, alcohol intake, ACEI/ARB intake, β-blocker intake, CCB intake, diuretic intake, 24 h mean SBP, 24 h mean DBP, Log 24 h-MAU/eGFR and UA. And the results showed that UA was significant related to Log 24 h-MAU in female patients (*β* = 0.269, *P* = 0.001) and eGFR both in male and female patients (male: *β* = − 0.175, *P* < 0.001; female: *β* = − 0.209, *P* = 0.003) (Table 5).

**Table 5:** The multivariate linear regression analysis for Log 24 h-MAU and eGFR in subgroup analysis

Gender	Variates	Log 24 h-MAU				Variates	eGFR			
		B	SE	*β*	*P*		B	SE	*β*	*P*
Male	UA (every increased 10 μmol/L)	0.004	0.002	0.087	0.060	UA (every increased 10 μmol/L)	− 0.518	0.138	− 0.175	< 0.001
	BMI	0.010	0.006	0.077	0.096	Age	− 0.681	0.130	− 0.249	< 0.009
	Duration of hypertension					ACEI/ARB intake	− 4.991	2.599	− 0.089	0.055
	Less than 1 year	Reference				24 h mean DBP	− 0.308	0.102	− 0.138	0.003
	1–5 years	0.029	0.050	0.030	0.565					
	5–10 years	0.159	0.062	0.127	0.010					
	More than 10 years	0.105	0.065	0.081	0.109					
	Grade 3 hypertension	0.170	0.044	0.176	< 0.001					
	CCB intake	0.094	0.043	0.099	0.027					
	Diuretic intake	0.126	0.068	0.081	0.065					
	24 h mean SBP	0.005	0.001	0.156	0.001					
	eGFR	− 0.002	0.001	− 0.105	0.017					
Female	UA (every increased 10 μmol/L)	0.015	0.004	0.269	0.001	UA (every increased 10 μmol/L)	− 0.855	0.286	− 0.209	0.003
	24 h mean SBP	0.007	0.002	0.232	0.003	Age	− 0.911	0.173	− 0.409	< 0.001
						ACEI/ARB intake	− 11.915	4.716	− 0.188	0.013

Multivariate analysis was adjusted for age, BMI, duration of hypertension, grade of hypertension, smoking, alcohol intake, ACEI/ARB intake, β-blocker intake, CCB intake, diuretic intake, 24 h mean SBP, 24 h mean DBP, Log 24 h-MAU/eGFR and UA.

*24 h-MAU*: 24-h microalbuminuria; *eGFR*: estimated glomerular filtration rate; *UA*: uric acid; *BMI*: body mass index; *ACEI/ARB*: angiotensin-converting enzyme inhibitor/angiotensin receptor inhibitor

In logistic regression analysis, the models enrolled age, BMI, duration of hypertension, grade of hypertension, ACEI/ARB intake, β-blocker intake, CCB intake, diuretic intake, fasting glucose, 2-h glucose, 24 h mean SBP, 24 h mean DBP, increased 24 h-MAU/mildly decreased eGFR and UA. The results showed that UA was significant related to increased 24 h-MAU (quantitative analysis: *OR* = 1.034, *95%CI* 1.003–1.065, *P* = 0.033 for male and *OR* = 1.084, *95%CI* 1.011–1.163, *P* = 0.023 for female; qualitative analysis: *OR* = 1.821, *95%CI* 1.078–3.076, *P* < 0.001 for male and *OR* = 3.741, *95%CI* 1.066–13.127, *P* = 0.039 for female), but showed inconspicuous relationship with mildly decreased eGFR in each gender (Table 6).

**Table 6:** The multivariate logistic regression analysis for increased 24 h-MAU and mildly decrease eGFR in subgroup analysis

Model*	Variates	Increased 24 h-MAU				Variates	Mildly decrease eGFR			
		B	SE	*P*	OR (95%CI)		B	SE	*P*	OR (95%CI)
1 (male)	UA (every increased 10 μmol/L)	0.033	0.016	0.033	1.034 (1.003–1.065)	Fasting glucose	0.785	0.219	< 0.001	2.193 (1.428–3.369)
	BMI	0.090	0.037	0.014	1.094 (1.018–1.175)	Diuretic intake	1.008	0.469	0.031	2.741 (1.094–6.868)
	Duration of hypertension			0.001						
	Less than 1 year	Reference								
	1–5 years	0.112	0.331	0.735	1.118 (0.585–2.138)					
	5–10 years	1.075	0.396	0.007	2.930 (1.349–6.363)					
	More than 10 years	1.170	0.394	0.003	3.223 (1.490–6.975)					
	Grade 3 hypertension	1.328	0.310	< 0.001	3.772 (2.056–6.923)					
1 (female)	UA (every increased 10 μmol/L)	0.081	0.036	0.023	1.084 (1.011–1.163)	Increased 24 h-MAU	2.238	1.255	0.075	9.375 (0.801–109.775)
	ACEI/ARB intake	− 1.432	0.613	0.019	0.239 (0.072–0.793)					
2 (male)	Hyperuricemia	1.336	0.309	< 0.001	1.821 (1.078–3.076)	Fasting glucose	0.785	0.219	< 0.001	2.193 (1.428–3.369)
	BMI	0.093	0.036	0.010	1.098 (1.023–1.178)	Diuretic intake	1.008	0.469	0.031	2.741 (1.094–6.868)
	Duration of hypertension			0.002						
	Less than 1 year	Reference								
	1–5 years	0.067	0.329	0.839	1.069 (0.561–2.035)					
	5–10 years	1.042	0.393	0.008	2.834 (1.311–6.125)					
	More than 10 years	1.141	0.392	0.004	3.130 (1.451–6.751)					
	Grade 3 hypertension	1.336	0.309	< 0.001	3.803 (2.074–6.974)					
2 (female)	Hyperuricemia	1.319	0.641	0.039	3.741 (1.066–13.127)	Increased 24 h-MAU	2.238	1.255	0.075	9.375 (0.801–109.775)
	ACEI/ARB intake	− 1.340	0.605	0.027	0.262 (0.080–0.857)					

*Model 1: included UA (every increased 10 μmol/L) as argument. Model 2: included hyperuricemia as argument. Multivariate analysis was adjusted for age, BMI, duration of hypertension, grade of hypertension, ACEI/ARB intake, β-blocker intake, CCB intake, diuretic intake, fasting glucose, 2-h glucose, 24 h mean SBP, 24 h mean DBP, increased 24 h-MAU/mildly decreased eGFR and UA.

*24 h-MAU*: 24-h microalbuminuria; *increased 24 h-MAU*: 24 h-MAU ≥ 30 mg/24 h; *eGFR*: estimated glomerular filtration rate; mildly decreased *eGFR*: eGFR < 90 ml ·min^−1^ ·1.73 m^−2^; *UA*: uric acid; *BMI*: body mass index; *ACEI/ARB*: angiotensin-converting enzyme inhibitor/angiotensin receptor inhibitor

## Discussion

In this study, the correlation between UA and kidney damage indicators 24 h-MAU and eGFR was investigated using hypertensive inpatients’ data, and the results indicated that UA had significant relationship with Log 24 h-MAU, eGFR and increased 24 h-MAU, but the association with mildly decreased eGFR was affected to a variety of confounding factors. And it was stable in the subgroup analysis for difference gender.

The Uric Acid Right for Heart Health (URRAH) Project was a large community populations study for estimating the relationship between UA and the risk of cardiovascular disease [10]. The results suggested a strong correlation among hyperuricemia, all-cause mortality (ACM), cardiovascular mortality (CVM), eGFR state, microalbumin and CKD [10, 11]. The result of receiver operator characteristic (ROC) curve showed that the UA cut-off value was 4.7 mg/dl for ACM, 5.6 mg/dl for CVM and 6.9 mg/dl for myocardial infraction [11]. And the UA cut-off value for CVM was 5.6 mg/dl for male and 5.1 mg/dl for female [12] A reanalysis was conducted to further clarify whether newly UA cut-off value for CVM was applicable to the hypertensive population in this study. The relationship among hyperuricemia [cut-off value for CVM = 5.6 mg/dl (333 μmol/L) for male and 5.1 mg/dl (303 μmol/L) for female], increased 24 h-MAU and eGFR was similar to the previous analysis (Additional file 1: Table S3). The UA cut-off values for increased 24 h-MAU was 450 μmol/L for male and 348 μmol/L for female, and the cut-off value for mildly decreased eGFR was 441 μmol/L for male and 394 μmol/L for female (Additional file 1: Table S4). Therefore, the UA cut-off value of 420 μmol/L for male and 360 μmol/L for female might be more suitable for patients enrolled in the present study [11].

In prehypertension or hypertension patients, some studies also indicated that UA was significantly associated with the increase of urinary microalbumin [13, 14] and the decrease of eGFR [15, 16]. The change of eGFR after UA-lowering treatment (ULT) might indirectly reflect the correlation between UA and eGFR. The previous study showed that eGFR levels were significantly higher after ULT, and the change of eGFR was negatively correlated with the change in UA levels [17]. A recent meta-analysis found that ULT might be useful for improvement of eGFR and reduction of urinary albumin/creatinine ratio in CKD patients [18]. The result was similar to many other meta-analysis [19–21].

In the present study, younger age of enrolled patients and lower prevalence of mildly decreased eGFR might be the major reason for the lack of association between UA and mildly decreased eGFR in the multivariate logistic regression. And highly ratio of ACEI/ARB intake (37.7%) might affect the evaluation of the association between UA and mildly decreased eGFR as well [22–24].

Existing studies had found that elevated UA could cause kidney damage by activation of the inflammatory system and RAAS system, renal interstitial fibrosis and increased vascular endothelial permeability, finally the clinical symptom showed as increased urinary albumin [25]. Glomerular filtration albumin increased the reuptake of excessive albumin by proximal tubular cells, leading to the activation of a variety of pathways and accelerating the damage of renal function [26]. In addition, urinary albumin-lowing treatment might be benefit to the risk of CKD. Every 30% reduction of urinary albumin might the decreased by 23.7% (*95%CI* 11.4–34.2%) for risk of end-stage renal disease [27]. And the results indicated the relationship between urinary albumin and CKD. In our study, the results of mediating effect analysis suggested that the increase of UA might cause the increased 24 h-MAU, and the subsequent increased 24 h-MAU caused the mildly decreased eGFR. However, this result could also be interpreted as different indicators in different stages of UA-mediated kidney damage. However, the causal relationship among the three could not be determined in the present study. More studies are still needed to explore the interaction mechanism among the three factors.

But we needed to pay special attention to those studies and meta-analysis which showed the negative conclusions. A study for the patients with different types of hypertensions found that UA was not significant associate to increased urinary microalbumin [28]. Another study just showed the relationship between UA and increased microalbumin, but not with creatinine clearance [13]. A ULT study for hypertensive patients with uncontrolled UA noticed that few changes in eGFR levels after UA-lowering therapy [29]. ULT could lowering the level of UA, but showed no benefit to renal disease in a meta-analysis [30]. Importantly, an umbrella review noticed that current evidence from retrospective study, randomized controlled trial, meta-analysis and mendelian randomization study could not fully demonstrate the role of uric acid in renal dysfunction [31]. Therefore, the relationship between UA and renal dysfunction and whether ULT improves renal function remains to be further explored.

This study also had the following limitations: The study was a retrospective study and could not determine the causal relationship among 24 h-MAU, eGFR and related factors. As the study was a single-center study, 24 h-MAU might be not widely available for each center and there might other factors influence eGFR, the conclusions might not be applicable to populations from other centers or regions. At last, repeated measurement for each date might improve the accuracy of the conclusions.

In conclusion, the prevalence of hyperuricemia was higher in hypertensive inpatients. UA was strongly associated with Log 24 h-MAU, eGFR and increased 24 h-MAU, while the correlation with mildly decreased eGFR was affected by multiple factors. And increased 24 h-MAU might be the intermediate factor between UA and the mildly decreased eGFR.

## Supplementary Information

**Additional file 1**: **Table S1**. The univariate linear regression analysis for Log 24h-MAU and eGFR. **Table S2**. The univariate logistic regression analysis for increased 24h-MAU and mildly decreased eGFR. **Table S3**. The logistic regression analysis for increased 24h-MAU and mildly decreased eGFR. **Table S4**. The ROC curve and UA cut-off value for increased 24h-MAU and mildly decreased eGFR.

## Data Availability

The datasets used and/or analyzed during the current study available from the corresponding author on reasonable request.

## Abbreviations

ACM: All-cause mortality
CKD: Chronic kidney disease
CVM: Cardiovascular mortality
eGFR: Estimated glomerular filtration rate
UA: Uric acid
ULT: Uric acid lowering therapy
24 h-MAU: 24-Hour microalbumin
