# Supplementary material for: The association among uric acid, microalbumin and estimated glomerular filtration rate in hypertensive patients: a case control study
Source: BMC Cardiovasc Disord. 2023 Feb 5;23:68. doi: 10.1186/s12872-023-03085-2 (PMC9899386; doi:10.1186/s12872-023-03085-2)
Supplement: Supplementary file 1 — Additional file 1: Table S1. The univariate linear regression analysis for Log 24h-MAU and eGFR. Table S2. The univariate logistic regression analysis for increased 24h-MAU and mildly decreased eGFR. Table S3. The logistic regression analysis for increased 24h-MAU and mildly decreased eGFR. Table S4. The ROC curve and UA cut-off value for increased 24h-MAU and mildly decreased eGFR. [file 12872_2023_3085_MOESM1_ESM.docx]

**Table S1** **The univariate linear regression analysis for Log 24h-MAU and eGFR**

| Variates | Log 24h-MAU | | | | |  | eGFR | | | | |
| --- | --- | --- | --- | --- | --- | --- | --- | --- | --- | --- | --- |
|  | *B* | *SE* | *β* | *t* | *P* |  | *B* | *SE* | *β* | *t* | *P* |
| Age | 0.001 | 0.001 | 0.020 | 0.532 | 0.595 |  | -0.543 | 0.088 | -0.224 | -6.202 | <0.001 |
| Gender (male) | 0.072 | 0.040 | 0.069 | 1.813 | 0.070 |  | -18.332 | 2.375 | -0.275 | -7.719 | <0.001 |
| BMI | 0.021 | 0.004 | 0.183 | 4.902 | <0.001 |  | -0.826 | 0.279 | -0.109 | -2.964 | 0.003 |
| Duration of hypertension |  |  |  |  |  |  |  |  |  |  |  |
| Less than 1 year |  |  | Reference | |  |  |  |  | Reference | |  |
| 1-<5 years | 0.053 | 0.042 | 0.005 | 1.265 | 0.206 |  | -4.962 | 2.675 | -0.079 | -1.855 | 0.064 |
| 5-<10 years | 0.191 | 0.053 | 0.151 | 3.596 | <0.001 |  | -5.453 | 3.330 | -0.067 | -1.637 | 0.102 |
| More than 10 years | 0.173 | 0.052 | 0.141 | 3.333 | 0.001 |  | -12.084 | 3.275 | -0.152 | -3.689 | <0.001 |
| Grade 3 hypertension | 0.235 | 0.035 | 0.244 | 6.627 | <0.001 |  | -5.605 | 2.297 | -0.090 | -2.440 | 0.015 |
| Smoking | 0.092 | 0.036 | 0.097 | 2.580 | 0.010 |  | -6.667 | 2.274 | -0.108 | -2.931 | 0.003 |
| Alcohol intake | 0.081 | 0.048 | 0.064 | 1.692 | 0.091 |  | -6.949 | 3.056 | -0.084 | -2.274 | 0.023 |
| Diabetes mellites | 0.169 | 0.070 | 0.091 | 2.417 | 0.016 |  | -0.162 | 4.461 | -0.001 | -0.036 | 0.971 |
| ACEI/ARB intake | -0.010 | 0.038 | -0.011 | -0.275 | 0.783 |  | -8.793 | 2.348 | -0.144 | -3.746 | <0.001 |
| β-blocker intake | -0.026 | 0.053 | -0.019 | 0.484 | 0.629 |  | -1.231 | 3.326 | -0.014 | -0.370 | 0.771 |
| CCB intake | 0.079 | 0.038 | 0.083 | 2.099 | 0.036 |  | -4.587 | 2.360 | -0.075 | -1.944 | 0.052 |
| Diuretic intake | 0.105 | 0.060 | 0.070 | 1.763 | 0.078 |  | -12.081 | 3.710 | -0.125 | -3.256 | 0.001 |
| α-blocker intake | -0.073 | 0.102 | -0.029 | -0.721 | 0.471 |  | -5.573 | 6.532 | -0.033 | -0.853 | 0.394 |
| MRA intake | 0.211 | 0.235 | 0.036 | 0.897 | 0.370 |  | -20.785 | 15.116 | -0.053 | -1.375 | 0.170 |
| WBC | 0.040 | 0.01 | 0.141 | 3.731 | <0.001 |  | 0.372 | 0.682 | 0.020 | 0.546 | 0.585 |
| RBC | 0.069 | 0.039 | 0.067 | 1.768 | 0.077 |  | -11.488 | 2.405 | -0.175 | -4.777 | <0.001 |
| HGB | 0.002 | 0.001 | 0.056 | 1.459 | 0.145 |  | -0.391 | 0.071 | -0.201 | -5.533 | <0.001 |
| Fasting glucose | 0.062 | 0.013 | 0.184 | 4.907 | <0.001 |  | 0.053 | 0.821 | 0.002 | 0.065 | 0.949 |
| 2-hour glucose | 0.027 | 0.007 | 0.167 | 3.728 | <0.001 |  | -0.054 | 0.488 | -0.005 | -0.110 | 0.913 |
| TC | 0.079 | 0.020 | 0.152 | 4.030 | <0.001 |  | -0.499 | 1.269 | -0.015 | -0.393 | 0.694 |
| TG | 0.047 | 0.013 | 0.134 | 3.548 | <0.001 |  | -1.876 | 0.857 | -0.081 | -2.188 | 0.029 |
| HDL-C | -0.055 | 0.071 | -0.030 | -0.777 | 0.437 |  | 15.578 | 4.424 | 0.130 | 3.521 | <0.001 |
| LDL-C | 0.084 | 0.022 | 0.144 | 3.815 | <0.001 |  | -0.894 | 1.411 | -0.024 | -0.634 | 0.526 |
| 24-hour urinary sodium | 0.000 | 0.000 | 0.067 | 1.770 | 0.077 |  | -0.006 | 0.018 | -0.014 | -0.357 | 0.721 |
| UA (every increased 10μmol/L) | 0.010 | 0.002 | 0.210 | 5.646 | <0.001 |  | -0.856 | 0.106 | -0.286 | -8.081 | <0.001 |
| daytime mean SBP, | 0.007 | 0.001 | 0.243 | 6.521 | <0.001 |  | -0.103 | 0.069 | -0.056 | -1.489 | 0.137 |
| nighttime mean SBP | 0.006 | 0.001 | 0.244 | 6.509 | <0.001 |  | -0.094 | 0.062 | -0.057 | -1.511 | 0.131 |
| 24h mean SBP | 0.007 | 0.001 | 0.257 | 6.902 | <0.001 |  | -0.106 | 0.070 | -0.057 | -1.508 | 0.132 |
| daytime mean DBP | 0.006 | 0.001 | 0.169 | 4.450 | <0.001 |  | -0.111 | 0.084 | -0.049 | -1.319 | 0.188 |
| nighttime mean DBP | 0.008 | 0.001 | 0.236 | 6.291 | <0.001 |  | -0.164 | 0.083 | -0.074 | -1.980 | 0.048 |
| 24h mean DBP | 0.007 | 0.001 | 0.191 | 5.053 | <0.001 |  | -0.130 | 0.086 | -0.056 | -1.499 | 0.134 |
| Log 24h-MAU | - | - | - | - | - |  | -10.018 | 2.435 | -0.154 | -4.113 | <0.001 |
| eGFR | -0.002 | 0.001 | -0.154 | -4.113 | <0.001 |  | - | - | - | - | - |

24h-MAU: 24-hour microalbuminuria; eGFR: estimated glomerular filtration rate; BMI: body mass index; ACEI/ARB: angiotensin-converting enzyme inhibitor/angiotensin receptor inhibitor; CCB: calcium channel blockers; MRA: mineralocorticoid receptor antagonists; WBC: white blood cell; RBC: red blood cell; HGB: hemoglobin; TC: total cholesterol; TG: triglycerides; SBP: systolic blood pressure; DBP: diastolic blood pressure.

**Table S2 The univariate logistic regression analysis for increased 24h-MAU and mildly decreased eGFR**

| Variates | Increased 24h-MAU | | | | | |  | Mildly decreased eGFR | | | | | |
| --- | --- | --- | --- | --- | --- | --- | --- | --- | --- | --- | --- | --- | --- |
|  | *B* | *SE* | *Wald χ^2^* | *P* | *OR* | *95%CI* |  | *B* | *SE* | *Wald χ^2^* | *P* | *OR* | *95%CI* |
| Age | 0.006 | 0.007 | 0.670 | 0.413 | 1.006 | 0.992~1.020 |  | 0.039 | 0.010 | 15.015 | <0.001 | 1.040 | 1.019~1.060 |
| Gender (male) | 0.538 | 0.206 | 6.840 | 0.009 | 1.713 | 1.114~2.564 |  | 0.902 | 0.391 | 5.334 | 0.021 | 2.465 | 1.146~5.302 |
| BMI | 0.107 | 0.022 | 24.343 | <0.001 | 1.113 | 1.067~1.161 |  | 0.074 | 0.033 | 5.204 | 0.023 | 1.077 | 1.011~1.148 |
| Duration of hypertension |  |  | 20.273 | <0.001 |  |  |  |  |  | 8.095 | 0.044 |  |  |
| Less than 1 year | Reference | | | | | |  |  | Reference | | | | |
| 1-<5 years | 0.071 | 0.220 | 0.103 | 0.748 | 1.073 | 0.697~1.651 |  | 0.633 | 0.402 | 2.485 | 0.115 | 1.884 | 0.857~4.139 |
| 5-<10 years | 0.836 | 0.252 | 10.974 | 0.001 | 2.307 | 1.407~3.782 |  | 0.953 | 0.444 | 4.603 | 0.032 | 2.592 | 1.086~6.189 |
| More than 10 years | 0.807 | 0.248 | 10.578 | 0.001 | 2.242 | 1.378~3.646 |  | 1.148 | 0.425 | 7.311 | 0.007 | 3.151 | 1.371~7.242 |
| Grade 3 hypertension | 1.196 | 0.205 | 33.943 | <0.001 | 3.308 | 2.121~4.948 |  | 0.776 | 0.335 | 5.381 | 0.020 | 2.173 | 1.128~4.186 |
| Smoking | 0.205 | 0.172 | 1.424 | 0.233 | 1.227 | 0.877~1.718 |  | 0.472 | 0.277 | 2.905 | 0.088 | 1.604 | 0.932~2.761 |
| Alcohol intake | 0.437 | 0.219 | 3.981 | 0.046 | 1.548 | 1.008~2.378 |  | 0.858 | 0.315 | 7.428 | 0.006 | 2.358 | 1.272~4.369 |
| Diabetes mellites | 0.713 | 0.310 | 5.282 | 0.022 | 2.040 | 1.111~3.746 |  | 0.574 | 0.460 | 1.558 | 0.212 | 1.776 | 0.721~4.376 |
| ACEI/ARB intake | -0.066 | 0.180 | 0.135 | 0.713 | 0.936 | 0.658~1.331 |  | 0.580 | 0.288 | 4.053 | 0.044 | 1.786 | 1.015~3.140 |
| β-blocker intake | -0.258 | 0.262 | 0.963 | 0.326 | 0.773 | 0.462~1.293 |  | 0.057 | 0.401 | 0.050 | 0.887 | 1.059 | 0.483~2.321 |
| CCB intake | 0.342 | 0.178 | 3.718 | 0.054 | 1.408 | 0.994~1.995 |  | 0.713 | 0.291 | 6.020 | 0.014 | 2.040 | 1.154~3.607 |
| Diuretic intake | 0.403 | 0.268 | 2.262 | 0.133 | 1.496 | 0.885~2.527 |  | 1.095 | 0.347 | 9.964 | 0.002 | 2.990 | 1.151~5.902 |
| α-blocker intake | 0.375 | 0.452 | 0.668 | 0.407 | 1.455 | 0.600~3.531 |  | 0.149 | 0.756 | 0.039 | 0.844 | 1.161 | 0.264~5.107 |
| MRA intake | -0.168 | 1.158 | 0.021 | 0.885 | 0.846 | 0.087~8.183 |  | 1.359 | 1.164 | 1.363 | 0.243 | 3.891 | 0.398~38.073 |
| WBC | 0.118 | 0.051 | 5.391 | 0.020 | 1.125 | 1.019~1.243 |  | 0.075 | 0.082 | 0.840 | 0.359 | 1.078 | 0.918~1.267 |
| RBC | 0.371 | 0.190 | 3.806 | 0.051 | 1.450 | 0.998~2.105 |  | 0.549 | 0.308 | 3.168 | 0.075 | 1.731 | 0.946~3.167 |
| HGB | 0.010 | 0.006 | 2.950 | 0.086 | 1.010 | 0.999~1.021 |  | 0.020 | 0.010 | 4.387 | 0.036 | 1.020 | 1.001~1.040 |
| Fasting glucose | 0.247 | 0.064 | 15.067 | <0.001 | 1.281 | 1.130~1.451 |  | 0.145 | 0.075 | 3.704 | 0.054 | 1.156 | 0.997~1.340 |
| 2-hour glucose | 0.103 | 0.036 | 8.320 | 0.004 | 1.109 | 1.034~1.190 |  | 0.106 | 0.052 | 4.109 | 0.043 | 1.111 | 1.004~1.231 |
| TC | 0.291 | 0.096 | 9.174 | 0.002 | 1.338 | 1.108~1.161 |  | 0.073 | 0.156 | 0.220 | 0.639 | 1.076 | 0.792~1.462 |
| TG | 0.165 | 0.062 | 7.199 | 0.007 | 1.180 | 1.046~1.332 |  | 0.078 | 0.096 | 0.667 | 0.414 | 1.081 | 0.896~1.305 |
| HDL-C | -0.333 | 0.325 | 1.046 | 0.306 | 0.717 | 0.379~1.357 |  | -0.499 | 0.590 | 0.717 | 0.397 | 0.607 | 0.191~1.928 |
| LDL-C | 0.309 | 0.107 | 8.348 | 0.004 | 1.363 | 1.105~1.681 |  | 0.042 | 0.176 | 0.056 | 0.813 | 1.042 | 0.739~1.471 |
| 24-hour urinary sodium | 0.002 | 0.001 | 1.995 | 0.158 | 1.002 | 0.999~1.004 |  | -0.002 | 0.002 | 0.678 | 0.410 | 0.998 | 0.994~1.003 |
| UA (every increased 10μmol/L) | 0.045 | 0.009 | 26.136 | <0.001 | 1.046 | 1.028~1.064 |  | 0.054 | 0.014 | 15.914 | <0.001 | 1.055 | 1.028~1.084 |
| Hyperuricemia | 0.693 | 0.172 | 16.223 | <0.001 | 2.000 | 1.427~2.803 |  | 0.865 | 0.278 | 9.662 | 0.002 | 2.374 | 1.376~4.095 |
| daytime mean SBP, | 0.025 | 0.005 | 21.087 | <0.001 | 1.025 | 1.014~1.036 |  | 0.019 | 0.009 | 5.171 | 0.023 | 1.020 | 1.003~1.037 |
| nighttime mean SBP | 0.019 | 0.005 | 16.401 | <0.001 | 1.019 | 1.010~1.029 |  | 0.019 | 0.007 | 7.244 | 0.007 | 1.019 | 1.005~1.034 |
| 24h mean SBP | 0.026 | 0.005 | 22.381 | <0.001 | 1.026 | 1.015~1.037 |  | 0.021 | 0.009 | 5.815 | 0.016 | 1.021 | 1.004~1.038 |
| daytime mean DBP | 0.024 | 0.007 | 12.507 | <0.001 | 1.024 | 1.011~1.038 |  | 0.011 | 0.011 | 0.982 | 0.322 | 1.011 | 0.990~1.033 |
| nighttime mean DBP | 0.028 | 0.007 | 19.043 | <0.001 | 1.029 | 1.016~1.042 |  | 0.020 | 0.010 | 3.738 | 0.053 | 1.020 | 1.000~1.041 |
| 24h mean DBP | 0.027 | 0.007 | 15.469 | <0.001 | 1.028 | 1.014~1.042 |  | 0.013 | 0.011 | 1.474 | 0.255 | 1.014 | 0.992~1.036 |
| Increased 24h-MAU | - | - | - | - |  | - |  | 1.137 | 0.289 | 15.433 | <0.001 | 3.116 | 1.767~5.494 |
| Mildly decreased eGFR | 1.137 | 0.289 | 15.433 | <0.001 | 3.166 | 1.767~5.494 |  | - | - | - | - | - | - |

24h-MAU: 24-hour microalbuminuria; increased 24h-MAU: 24h-MAU≥30mg/24h; eGFR: estimated glomerular filtration rate; mildly decreased eGFR: eGFR<90ml·min^-1^·1.73m^-2^; BMI: body mass index; ACEI/ARB: angiotensin-converting enzyme inhibitor/angiotensin receptor inhibitor; CCB: calcium channel blockers; MRA: mineralocorticoid receptor antagonists; WBC: white blood cell; RBC: red blood cell; HGB: hemoglobin; TC: total cholesterol; TG: triglycerides; UA: uric acid; SBP: systolic blood pressure; DBP: diastolic blood pressure.

**Table S3 The logistic regression analysis for increased 24h-MAU and mildly decreased eGFR**

|  | Variates | Increased 24h-MAU | | | | Variates | Mildly decrease eGFR | | | |
| --- | --- | --- | --- | --- | --- | --- | --- | --- | --- | --- |
|  |  | *B* | *SE* | *P* | *OR (95%CI)* |  | *B* | *SE* | *P* | *OR (95%CI)* |
| Univariate | Hyperuricemia (newly cut-off) | 0.708 | 0.201 | <0.001 | 2.029 (1.369~3.007) | Hyperuricemia (newly cut-off) | 1.056 | 0.390 | 0.007 | 2.876 (1.339~6.179) |
| Multivariate | Hyperuricemia (newly cut-off) | 1.008 | 0.325 | 0.002 | 2.711 (1.451~5.179) | Age | 0.050 | 0.021 | 0.018 | 1.051 (1.009~1.096) |
|  | Duration of hypertension |  |  | 0.043 |  | RBC | 1.023 | 0.451 | 0.023 | 2.783 (1.149~6.741) |
|  | Less than 1 year | Reference | | | | Fasting glucose | 0.468 | 0.230 | 0.042 | 1.597 (1.017~2.509) |
|  | 1-<5 years | 0.044 | 0.299 | 0.883 | 1.045 (0.581~1.878) | Nighttime mean DBP | 0.028 | 0.014 | 0.038 | 1.029 (1.002~1.057) |
|  | 5-<10 years | 0.823 | 0.352 | 0.020 | 2.276 (1.141~4.541) | Increased 24h-MAU | 0.705 | 0.390 | 0.071 | 2.023 (0.942~4.343) |
|  | More than 10 years | 0.601 | 0.359 | 0.094 | 1.824 (0.903~3.683) |  |  |  |  |  |
|  | Grade 3 hypertension | 0.980 | 0.277 | <0.001 | 2.663 (1.547~4.585) |  |  |  |  |  |
|  | Fasting Glucose | 0.358 | 0.159 | 0.024 | 1.430 (1.047~1.952) |  |  |  |  |  |
|  | Mildly decreased eGFR | 0.795 | 0.398 | 0.046 | 2.214 (1.014~4.834) |  |  |  |  |  |

Hyperuricemia (newly cut-off): the UA cut-off value for cardiovascular mortality=5.6mg/dl for male and 5.1mg/dl for female; 24h-MAU: 24-hour microalbuminuria; increased 24h-MAU: 24h-MAU≥30mg/24h; eGFR: estimated glomerular filtration rate; mildly decreased eGFR: eGFR<90ml·min^-1^·1.73m^-2^; RBC: red blood cell; HGB: hemoglobin; DBP: diastolic blood pressure.

**Table S4 The ROC curve and UA cut-off value for increased 24h-MAU and mildly decreased eGFR**

|  | Male | | | | | |  | Female | | | | | |
| --- | --- | --- | --- | --- | --- | --- | --- | --- | --- | --- | --- | --- | --- |
|  | AUC | *P* | *95%CI* | Cut-off value | Sensitivity | Specificity |  | AUC | *P* | *95%CI* | Cut-off value | Sensitivity | Specificity |
| Increased 24h-MAU | 0.587 | 0.002 | 0.533~0.641 | 450μmol/L | 41.9% | 74.3% |  | 0.604 | 0.048 | 0.499~0.709 | 348μmol/L | 36.8% | 81.3% |
| Mildly decreased eGFR | 0.591 | 0.035 | 0.502~0.680 | 441μmol/L | 51.0% | 68.3% |  | 0.829 | 0.002 | 0.700~0.957 | 394μmol/L | 62.5% | 91.8% |

24h-MAU: 24-hour microalbuminuria; increased 24h-MAU: 24h-MAU≥30mg/24h; eGFR: estimated glomerular filtration rate; mildly decreased eGFR: eGFR<90ml·min^-1^·1.73m^-2^; UA: uric acid; AUC: area under the curve
